# Advance telephone calls ahead of reminder questionnaires increase response rate in non-responders compared to questionnaire reminders only: The RECORD phone trial

**DOI:** 10.1186/1745-6215-15-13

**Published:** 2014-01-08

**Authors:** Graeme MacLennan, Alison McDonald, Gladys McPherson, Shaun Treweek, Alison Avenell

**Affiliations:** 1 Health Services Research Unit, University of Aberdeen, Aberdeen, UK

**Keywords:** Telephone reminders, Postal questionnaires, Response rates, Average treatment effect on the treated

## Abstract

**Background:**

Postal questionnaires are simple and economical for collecting outcome data for randomised controlled trials (RCTs) but are prone to non-response. In the RECORD trial (a large pragmatic publicly funded RCT in UK) non-responders were sent a reminder and another questionnaire at 1 year, of which 40% were returned. In subsequent years we investigated the effect of an advance telephone call to non-responders on responses rate to reminder questionnaires and the next questionnaire 4 months later.

**Methods:**

Non-responders to annual questionnaires were randomised to receive a telephone call from the trial office ahead of the reminder questionnaire in addition to the usual reminder schedule (*n* = 390) or to a control group that received the usual reminder schedule only (*n* = 363). The primary outcome was response to the reminder questionnaire within 21 days; secondary outcomes were response to a questionnaire 4 months later; completeness of quality of life instruments; and the number of participants declining further follow-up. Results are presented as odds ratios from a logistic regression intention-to-treat (ITT) analysis and then percentage difference and 95% confidence intervals (CI) for both ITT and average treatment effect on the treated (ATT) analyses.

**Results:**

The proportions that responded were 67.8% (265/390) in the intervention group compared to 62.5% (227/363) in the control group. The ITT estimate was a 5.4% increase (95% CI −1.4 to 12.2). Four months later percentages responding were 51.8% (202) and 42.7% (155). The ITT estimate was a 9.1% increase (95% CI 2.0 to 16.2). In the intervention group 12.3% (48/390) of participants were not telephoned because questionnaires were returned before the scheduled telephone call. ATT estimates adjusting for this were 6.2% (95% CI −1.6 to 14.0) and 10.4% (95% CI 2.2 to 18.5), respectively.

**Conclusions:**

The telephone call resulted in a slight increase in response to the reminder questionnaire, however at 4 months later the proportion in the telephoned group responding was greater. This study suggests that pre-notification telephone calls may only be worthwhile if further questionnaires are to be sent out soon after reminder questionnaires.

**Trial registration:**

Current Clinical Trials ISRCTN51647438

## Background

Postal questionnaires are simple and economical for collecting outcome data for randomised controlled trials (RCTs) but achieving high response rates can be very challenging. The pitfalls of poor response rates are well documented and include loss of outcome data, reducing the effective sample size (and hence precision) of the trial and increasing the potential for bias [1].

Several strategies have been used to try to maximise response rates to research studies, including pre-notification and the use of incentives, and these have been widely reported. For example, the Cochrane Review by Edwards and colleagues looking at methods to increase response rates to postal and electronic questionnaires [2] identified 47 trials investigating the effect of pre-notification (that is, telling participants that they would receive a questionnaire soon). Pre-notification significantly increased response rates (odds ratio (OR) 1.45; 95% confidence interval (CI) 1.29 to 1.63) with probably little or no difference between contacting participants by telephone or by post (OR = 1.18; 95% CI 0.77 to 1.80).

The RECORD trial (Randomised Evaluation of Calcium and/OR vitamin D) involved people aged 70 years or over who had a previous fracture. RECORD participants were sent questionnaires every 4 months that recorded data on self-reported fracture and quality of life [3]. Annual questionnaires collected additional health economic outcomes and concomitant medication information. Non-responders were sent another questionnaire after 3 weeks. While preparing the report for the first data monitoring and safety committee it was noted that response rates were lower than required. A total of 397 (69%) of the 573 participants randomised who had reached 12-months follow-up by 30 September 2000 responded to the first annual questionnaire. Reminder questionnaires were not sent due to loss to follow-up (for example, trial office not notified of change of address), participants declining further follow-up, or notification of deaths. Of the 176 non-responders, 150 were mailed a reminder questionnaire and 62/150 (40%) responded. This yielded an extra 11% in response, increasing the overall proportion responding to 80% (459/573). This retention rate might be considered acceptable for many trials, however the primary event (fractures) in our RECORD trial population was expected to be relatively uncommon at 15% in the control group, making a very high response rate essential.

We were already telephoning participants that did not respond within 3 weeks of their reminder questionnaire, which was well-received by these participants and resulted in an extra 41 responses. It was unclear whether the addition of a telephone call to participants prior to sending the questionnaire (that is, pre-notification) would increase this further. We therefore embedded a methodological trial, the Phone Trial, of pre-notification telephone calls by randomly allocating non-responding participants to receive a pre-notification telephone call, or to receive RECORD’s usual reminder schedule.

## Methods

Participants in the RECORD trial who did not respond to the initial mailing of the annual questionnaire at 12- and 24-month follow-up, and were not randomised in other ongoing methodological sub-studies were considered eligible for the Phone Trial. Eligible participants were stratified by their RECORD trial allocation and randomised using a computerised central allocation process at the Trial Office to one of the following:

–The intervention group: Telephone call from the Trial Office up to 14 days before the questionnaire was sent, asking the participant to complete all the questions on the questionnaire when it arrived. This was followed by the usual RECORD reminder schedule (see below).

Or

–The control group (usual RECORD reminder schedule): Repeat mailing of usual follow-up letter and the questionnaire, followed by a phone call by the study nurse or the Trial Office if the questionnaire was not returned within 3 weeks.

The primary outcome of the Phone Trial was the proportion of reminder questionnaires returned within 3 weeks of the reminder being sent. Secondary outcomes were proportion of questionnaires returned at the next time-point 4 months later (that is, the 16-month or 20-month questionnaire); completeness of data for the EQ-5D and SF-12 quality of life measures (QoL) in annual questionnaires; and the number of participants declining further follow-up. We were interested in measuring the completeness of the QoL measures as these were secondary outcomes collected from everyone (the primary outcome was a rare outcome whose completeness could not be assessed) in the RECORD trial, in case people were more likely to return incomplete questionnaires. We also thought that the phone call might offer participants an extra opportunity to decline further follow-up so this potentially negative impact was also of interest.

### Statistical analysis

The sample size available was constrained by the number of eligible participants in the RECORD trial and a formal sample size calculation was not used to derive a target sample size for the Phone Trial.

**Figure 1:**
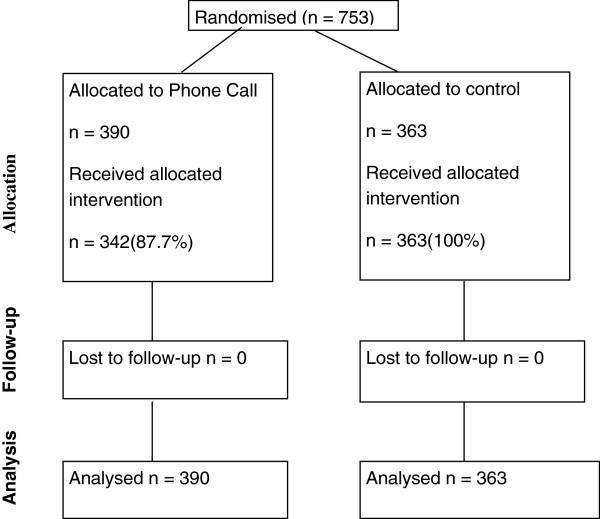
CONSORT Diagram.

If a participant was randomised more than once (that is, non-response to both 12- and 24-month follow-up questionnaires) only the first year response data were used. Descriptive data on the baseline characteristics are summarised using means and standard deviations (SD) and counts and percentages, where appropriate. All outcomes were analysed using logistic regression and effect sizes are presented as both ORs and absolute percentage differences (with 95% CIs) to aid interpretation. Analyses were by ITT, that is all participants were included regardless of whether they received the planned phone call or not. In the intervention group there were participants who did not receive the phone call (see Figure 1 for CONSORT diagram and Results below), mainly because they returned the original questionnaire before the reminder schedule initiated the call. We therefore carried out extra analyses to estimate the average treatment effect on the treated (ATT), that is, the effect of receiving a pre-notification telephone call. This analysis was carried out within an instrumental variables framework [4]. We also used a time-to-event analysis to assess the possibility that the pre-notification telephone call could harm response rates by giving participants an extra opportunity to decline further follow-up. Time to declining further follow-up was compared between the two groups using Cox regression and plotted using Kaplan-Meier plots. All analyses were done in Stata12. [StataCorp. 2011. Stata Statistical Software: Release 12. College Station, TX, USA: StataCorp LP].

### Ethical approval and consent

The RECORD trial was based in 21 hospitals in the UK. Ethics approval was obtained from the Multicentre Research Ethics Committee for Scotland and from the local research ethics committee of each hospital, and participants gave written informed consent.

## Results

Between 27 July 2001 and 7 January 2003 there were 753 RECORD participants randomised to the Phone Trial, 390 were allocated to the intervention group and 363 to the control group. The groups were well balanced at baseline (Table 1). In the intervention group 48 (12.3%) did not receive the phone call as planned, mainly because the questionnaire was returned within 3 weeks making the call unnecessary (Figure 1).

**Table 1:** RECORD trial baseline characteristics by Phone Trial allocated group

	**Phone call**	**Control**
	***n*** **= 390**	***n*** **= 363**
Age, mean (standard deviation)	77 (6)	77 (6)
≥ 80 years	117 (30)	112 (31)
Female	333 (85)	308 (85)
Type of enrolling fracture		
Proximal femur	62 (16)	56 (15)
Distal forearm	136 (35)	122 (34)
Other fracture	192 (49)	185 (51)
Time since enrolling fracture		
Within previous 3 months	326 (84)	299 (82)
More than previous 3 months	64 (16)	64 (18)
Baseline quality of life, mean (standard deviation)		
EQ-5D utility	0.708 (0.264)	0.697 [0.295]
SF-12 - Physical	40.2 (10.8)	40.2 (11.2)
SF-12 - Mental	50.0 (10.2)	48.8 (10.6)

Cells are number (%) unless otherwise indicated.

For the primary outcome, the number returning a questionnaire within 3 weeks of the first reminder in the intervention group was 265/390 (67.8%) compared to 227/363 (62.5%) in the control group. The ITT estimate of the between-group difference was a 5.4% increase (95% CI −1.4% to 12.2%) in the intervention group. The ATT estimate, which accounted for the 48 participants in the intervention group who did not need to receive the phone call, was 6.2% (95% CI −1.6%, 14.0%) (Table 2).

**Table 2:** Primary and secondary outcomes

	**Phone call**		**Control**		**ITT analyses**		**ATT analyses**
	***n*** **= 390**		***n*** **= 363**				
Outcome	*n*	(%)	*n*	%	OR (95% CI); *P*	RD (95% CI)	RD (95% CI); *P*
Response	265	(68.0)	227	(62.5)	1.27 (0.94, 1.72); 0.12	5.4 (−1.4, 12.2)	6.2 (−1.6, 14.0); 0.12
Completed EQ-5D	228	(58.5)	200	(55.1)	1.15 (0.85, 1.53); 0.35	3.4 (−3.7, 10.4)	3.8 (−4.3, 11.9); 0.35
Completed SF-12	208	(53.3)	187	(51.5)	1.08 (0.81, 1.43); 0.62	1.8 (−5.3, 9.0)	2.1 (−6.1, 10.2); 0.62
Response 4 months later	202	(51.8)	155	(42.7)	1.44 (1.08, 1.92); 0.013	9.1 (2.0, 16.2)	10.4 (2.2, 18.5); 0.013

ATT = average treatment effect on the treated; ITT = intention-to-treat; OR = odds ratio; RD = risk difference.

**Figure 2:**
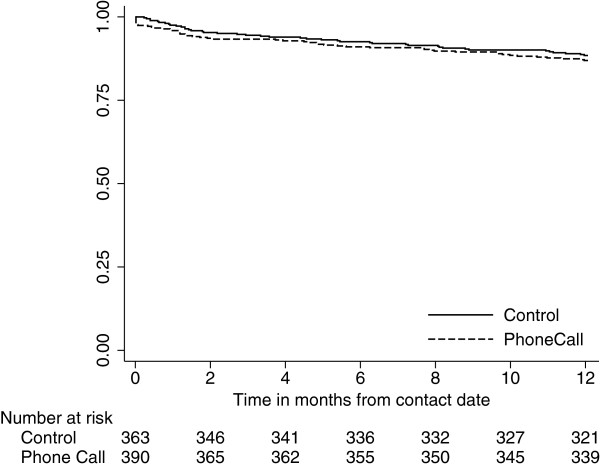
**Time to decline further follow-up within 12 months of contact.** The hazard ratio comparing the intervention to control was 1.14 (95% CI 0.76, 1.73).

There were participants in both intervention and control groups who returned questionnaires but did not complete the QoL sections with enough data to derive summary E5-QD utilities or SF-12 scores. The differences between groups in proportion completing the QoL sections were 3.4% (−3.7%, 10.4%) and 1.8% (−5.3%, 9.0%) for EQ-5D and SF-12, respectively, with both results being higher in the intervention group. There were nine intervention group participants who withdrew on the day they received their phone call. However, there was no evidence of an increase in the number of participants declining further follow-up (Figure 2), the hazard ratio was 1.14 (95% CI 0.76, 1.73).

## Discussion

In this study we found no conclusive evidence that a pre-notification phone call increased the proportion of trial participants responding to a reminder questionnaire when compared to the standard reminder schedule used within the RECORD trial. There was a small but perhaps worthwhile, increase of 5.4% (95% CI −1.4%, 12.2%) but the uncertainty around this estimate does not rule out the possibility that the intervention may actually harm response rates.

There are a number of possible reasons for this. First, the control group response rate at 62.5% was higher than anticipated. When the Phone Trial was first conceived only 40% of participants were responding to reminders. There was therefore a potential ceiling effect, with the maximum achievable response rate having already been reached, or almost reached, because of other response rate initiatives that were ongoing prior to the Phone Trial. Second, the 3-week cutoff for response may have been too short. This was chosen because a further reminder strategy (phone calls) was implemented within the RECORD trial for persistent non-responders after this time period had elapsed. Third, there was no pre-specified power calculation. Methodological studies to improve response rates are constrained by size of the parent trial in which they are embedded. If we consider the observed effect in the Phone Trial, a small but potentially worthwhile increase in response of 5.4%, then the sample size required (α = 5%; β = 90%) would be approximately 4,000 participants. Sample sizes of this order are hard to achieve for embedded methodological trials so it is essential that they are published, with clear methods and intervention descriptions, so that meta-analysis is possible.

We had three *a priori* concerns about the Phone Trial intervention. First, there was a concern that although responses may increase this would come at the expense of partial completion of QoL instruments, leading to unusable data. This is a possibility; for example, the proportion of the 265 responding to the reminder that had usable EQ5D data was 86% (228/265) in the Phone Call group, this proportion was 88% (200/227) in the control group. Second, there was a concern that the participants might use the pre-notification phone call as an opportunity to decline further follow-up. There were only nine individuals in the intervention group that did this; for the next 12 months the groups had very similar patterns of declining further follow-up. Third, we anticipated that due to the scheduling logistics of making the phone calls that some participants may not actually receive phone calls. We accounted for this in our analysis by using an ATT estimate in addition to the standard ITT estimate. In fact the intervention was well implemented and the results of the ITT and ATT analyses are similar.

The review by Edwards et al. [2] estimated the effect of pre-notification on final response as an OR of 1.45 (95% CI 1.29, 1.63). At first glance our results appear inconsistent with Edwards et al. However, their review found considerable heterogeneity between studies. We calculated the predictive distribution of a future trial (that is, the interval in which the treatment effect of a new trial might lie) based on the observed heterogeneity from Edwards et al. The estimated 95% predictive interval was 0.69 to 3.03; the estimate from the Phone Trial is entirely consistent with this. It is also worth noting that the Edwards’ review did not specifically focus on response to questionnaires used to collect outcome data for trials, which is the subject of a future Cochrane review [5]. We also investigated the effect of the intervention on the subsequent trial questionnaire, scheduled for 4 months after the annual questionnaires. There was a residual carry over into the next time period (see Table 2), with the response proportion to the subsequent questionnaire being higher in the Phone Call group. A limitation of this study is that we did not record either the number of phone calls to each participant before contact was made, or the duration of phone calls once contact had been made. An economic analysis to estimate the cost of the intervention was not possible. Researchers planning to trial such interventions in the future should record these data as the trade-off between cost and effectiveness is essential to assess the use of pre-notification phone calls.

## Conclusions

The telephone call resulted in a slight increase in response to the reminder questionnaire, however at 4 months later the proportion in the telephoned group responding was greater. This study suggests that pre-notification telephone calls may only be worthwhile if further questionnaires are to be sent out soon after reminder questionnaires.

## Competing interests

The authors declare that they have no competing interests.

## Authors’ contributions

GMac analysed and interpreted the data, and drafted and revised the manuscript. AM conceived the idea for the study, managed data collection and delivery of the intervention, interpreted the data, and drafted and revised the manuscript. GMc conceived the idea for the study, managed data collection, analysed and interpreted the data, and drafted and revised the manuscript. ST interpreted the data, and drafted and revised the manuscript. AA conceived the idea for the study, managed data collection and delivery of the intervention, interpreted the data, and drafted and revised the manuscript. All authors read and approved the final manuscript.
